# Hatred Is a Mindset Triggered by Stressful External Events, Negative Personal or Group Interpretations and Unhealthy Social Environments

**DOI:** 10.1192/bjo.2024.245

**Published:** 2024-08-01

**Authors:** Salman Shafiq

**Affiliations:** Lancashire and South Cumbria NHS Foundation Trust, Preston, United Kingdom

## Abstract

**Aims:**

To carry out systematic literature search on an international medical database to find what the emerging categories in which the word hatred is used in medical literature are, and to gather information regarding the generation of the emotion of hatred in human beings by thematically analysing the relevant collected data.

**Methods:**

To identify the information on hatred relevant for mental health professionals, we performed a systematic review using a systematic approach and criteria.

**Results:**

Six themes regarding generation of hatred identified.

Theme one: Targets of hatred.

Theme two: Self-hatred.

Theme three: Self-perceived hatred.

Theme four: Hatred towards inanimate objects.

Theme five: Reasons for hating other humans.

Theme six: Internal reasons for development of hatred.

**Conclusion:**

The word ‘hatred’ is used in medical literature in a multiplicity of meanings that range from using it in its literal sense to describe a subtle attitude such as a phobia-philia relationship, or to describe a unique outcome that is generated as an interplay of several different kind of factors. These may include cognitions, behaviours, social interactions, attitudes, sentiments, developmental backgrounds, psychodynamic interactions with others in real and virtual worlds etc. Hatred is more like a mind-set that people can develop towards themselves, towards others and towards inanimate objects or situations too. Fear, anger and disgust are primary emotions (that we are born with); human psyche is naturally prone to several inevitable cognitive errors; human thought is subjected to unavoidable logical fallacies; and human ego cannot avoid utilising unhealthy ego-defence mechanisms. Every child is born in a family and culture that has its own unique background and history. We humans are prone to the generation of the hateful mindset as an unavoidable outcome in a variety of scenarios. Keeping these generational patterns in view, it would be reasonable to say that an early detection and addressing the early warning signs towards development of the hateful mind-set would be helpful for ourselves and for others. As the word is used in several different meanings, the background information, context, and overall scenario of the discussion needs to be kept in mind whilst attempting to draw any meanings about the use of hate/hatred in a verbal or written expression. In each case where the word ‘hatred’ is used, needs to be approached with epistemic curiosity and in some instances, it may need detailed epistemic inquiry to fully comprehend the meaning of this word in any given expression.

